# Information Structure Influences Depth of Syntactic Processing: Event-Related Potential Evidence for the Chomsky Illusion

**DOI:** 10.1371/journal.pone.0047917

**Published:** 2012-10-24

**Authors:** Lin Wang, Marcel Bastiaansen, Yufang Yang, Peter Hagoort

**Affiliations:** 1 Key Laboratory of Behavioral Science, Institute of Psychology, Chinese Academy of Sciences, Beijing, China; 2 Max Planck Institute for Psycholinguistics, Nijmegen, The Netherlands; 3 Donders Institute for Brain, Cognition and Behaviour, Radboud University Nijmegen, Nijmegen, The Netherlands; University of Bern, Switzerland

## Abstract

Information structure facilitates communication between interlocutors by highlighting relevant information. It has previously been shown that information structure modulates the depth of semantic processing. Here we used event-related potentials to investigate whether information structure can modulate the depth of syntactic processing. In question-answer pairs, subtle (number agreement) or salient (phrase structure) syntactic violations were placed either in focus or out of focus through information structure marking. P600 effects to these violations reflect the depth of syntactic processing. For subtle violations, a P600 effect was observed in the focus condition, but not in the non-focus condition. For salient violations, comparable P600 effects were found in both conditions. These results indicate that information structure can modulate the depth of syntactic processing, but that this effect depends on the salience of the information. When subtle violations are not in focus, they are processed less elaborately. We label this phenomenon the Chomsky illusion.

## Introduction

During communication, people tend to organize their utterances so as to highlight the most relevant information. This way of linking new and/or important information with previously given information is referred to as information structure (IS) [1]. It divides a sentence into two parts: background and focus (for a review see [2]). Background refers to the information that is shared by the interlocutors, while focus refers to the information that is new or important to the listener/reader. For instance, in the question-answer-pair *Who orders a taxi after the party? The* ***guest*** *orders a taxi after the party*, the wh-question (*who*) inquired about specific information concerning the subject noun of the answer sentences. Accordingly, the constituent of the answer (the word in boldface) corresponding to the wh-word in the question conveys important information and thus has a focus status, while the other part of the answer refers to information already stated in the question, and hence forms the background [3].

Behavioral studies (including reaction time and eye-tracking studies) suggest that focused information is processed more deeply than non-focused information [4], [5], [6], [7]. This was further supported in a series of ERP studies testing the online processing of IS markers. These studies focused mainly on the N400. The N400 is a negative-going brain potential with a centro-posterior distribution. N400 effects are usually seen between 300 ms and 500 ms, and are often observed to the violation of semantic constraints (for a review, see [8]). Nevertheless, some studies also observed N400 effects in response to syntactic violations [9], [10], [11], which might be due to the difficulties of semantic integration as a consequence of a syntactic violation. In two recent ERP studies [12], [13], we investigated how IS influences the N400 effect in response to the semantic incongruency of a word’s meaning in relation to its context. The results showed that the semantic incongruency evoked a significantly larger N400 effect for focused than for non-focused information, which confirms the role of IS in modulating the depth of semantic processing. The current study addresses whether IS also has an influence on the depth of syntactic processing.

The P600/SPS (syntactic positive shift) is an ERP component that has been associated with syntactic processing [10], [14]. It has a posterior distribution, and occurs between around 500 ms and 1200 ms post-stimulus. P600 effects are usually reported in response to syntactic violations [10], [14], but are also elicited by syntactic ambiguity [15], and semantic reversal anomalies (for reviews see [16], [17]). Therefore, we can measure online syntactic processing by examining specific ERP responses to syntactic violations.

Gunter and Friederici [18] investigated the ERP responses to different levels of syntactic processing. They instructed the subjects either to judge the grammaticality of the sentences (which requires more detailed syntactic analysis) or to judge the printed cases (upper case or lower case) of particular words in the sentences (which induces only shallow syntactic processing). They found that compared to the grammaticality judgment task, the physical judgment task reduced the N400 and P600 effects elicited by the syntactic violations. The results suggested shallow processing might attenuate the amplitude of language related ERP effects.

In this study, we use IS to direct attentional resources towards, or away from, target words in sentences. When target words are in focus position, we hypothesize that the syntactic markers of these words will be processed deeply. When target words are in non-focus position, on the other hand, we hypothesize that the syntactic aspects of those words are processed in a more shallow fashion. ERPs will be recorded in response to syntactic violations that are either in focus or non-focus position. The ERP effects in response to syntactic violations will be compared between the focus and non-focus conditions. We hypothesize attenuated ERP effects in the non-focus condition compared to the focus condition.

More specifically, we manipulated IS by using wh-question-answer pairs, such that a critical word in the answer sentence was either in focus or in non-focus position. In addition, the grammaticality of the focused or non-focused constituent was manipulated. In order to explicitly examine the extent to which IS modulates syntactic processing, two types of grammatical violations were included. A number agreement violation violates the syntactic constraints in a subtle way, since the violated and correct words are often similar at the orthographic level (e.g. *order* vs. *orders*). In addition, we also constructed a more salient violation, a phrase structure violation, which is more likely to be detected than the number agreement violation. Based on previous studies [12], [13], we hypothesize that readers allocate more attentional resources to focused information and process it more deeply than non-focused information, resulting in larger P600 effects for the focused than non-focused information in response to syntactic violations. Nevertheless, this IS modulation might be overridden by the salience of the violation (e.g. phrase structure violation), resulting in similar P600 effects between focused and non-focused information for the phrase structure violations.

## Methods

### Participants

Twenty-four healthy native speakers of Dutch (12 females, mean age 20, range 18–26 years) were paid to participate in the experiment. They were all right handed, with neither dyslexia nor neurological abnormalities. A consent form according to the Declaration of Helsinki was signed before they started the experiment. The experiments were approved by the local ethics committee (Commissie Mensgebonden Onderzoek Regio Arnhem-Nijmegen).

### Stimuli

Question-answer pairs served as the experimental stimuli. The materials contained two different factors: Grammaticality and Context. In the question-answer pairs, all the answers had a fixed structure (See Table 1 for examples), in which the Grammaticality (Correct, Number agreement violation, Phase structure violation) was manipulated. In the correct condition, all the sentences were both syntactically and semantically correct (Table 1: the answer sentences in conditions 1 and 4). In the other two conditions, a number agreement violation or a phrase structure violation was created that became clear at the subject nouns. The number agreement violation was a combination of a singular verb and a plural subject (Table 1: between *bestelt (orders)* and *gasten (guests)* in the answer sentences in conditions 2 and 5). We only used the violation of “singular verb + plural subject” in order to make sure that the number agreement violation really occurred on the critical word (CW, subject noun), since for the opposite construction there is an alternative, syntactically correct continuation possible (plural verb + singular subject NP1+ singular subject NP2). The other syntactic violation, i.e., the phrase structure violation, became clear at the subject noun that was preceded by transposition of adverbs and adjectives (Table 1: *boze nogal gasten (angry rather guests)* in the answer sentences in conditions 3 and 6). Since the “adjective + adverb” combination could in principle be a part of the structure “adjective + adverb + adjective + noun”, e.g. *boze nogal dronken gasten (angry rather drunk guests),* it was only at the point of the subject noun that the sentence can no longer be continued in a grammatically well-formed manner. However, the reader already experienced parsing difficulties at the adverb following the adjective, because the “adjective + adverb + adjective + noun” structure is relatively infrequent and complex compared to the preferred “adverb + adjective + noun” sequence. Therefore, in addition to the CW (subject noun), we also analyzed the word preceding the CW (CW-1) for the phrase structure violation condition**,** as a P600 effect for the CW-1 has been reported before [10].

**Table 1:** An example of the six conditions for one experimental item set.

1. Focus, CorrectQuestion:*Wie bestelt er een taxi na het feest?* * (Who orders a taxi after the party?)*Answer:*Na afloop van het feest bestellen de nogal boze * ***gasten*** * een taxi.* * (After the party order the rather angry * ***guests*** * a taxi.)* * (The rather angry * ***guests*** * order a taxi after the party.)*2. Focus, Number agreement violationQuestion:*Wie bestelt er een taxi na het feest?* * (Who orders a taxi after the party?)*Answer:*Na afloop van het feest bestelt de nogal boze ***gasten*** * een taxi.* *(After the party orders the rather angry ***guests*** * a taxi.)* *(The rather angry ***guests*** * orders a taxi after the party.)*3. Focus, Phrase structure violationQuestion:*Wie bestelt er een taxi na het feest?* * (Who orders a taxi after the party?)*Answer:*Na afloop van het feest bestellen de boze nogal ***gasten*** * een taxi.* *(After the party order the angry rather ***guests*** * a taxi.)* *(The angry rather ***guests*** * order a taxi after the party.)*4. Non-focus, CorrectQuestion:*Wanneer bestelt men een taxi?* * (When does one order a taxi?)*Answer:***Na afloop van het feest*** * bestellen de nogal boze gasten een taxi.* * (* ***After the party*** * order the rather angry guests a taxi.)* * (The rather angry guests order a taxi * ***after the party*** *.)*5. Non-focus, Number agreement violationQuestion:*Wanneer bestelt men een taxi?* * (When does one order a taxi?)*Answer:* ***Na afloop van het feest*** * bestelt de nogal boze gasten een taxi.* *( ***After the party*** * orders the rather angry guests a taxi.)* *(The rather angry guests orders a taxi ***after the party*** *.)*6. Non-focus, Phrase structure violationQuestion:*Wanneer bestelt men een taxi?* * (When does one order a taxi?)*Answer:* ***Na afloop van het feest*** * bestellen de boze nogal gasten een taxi.* *( ***After the party*** * order the angry rather guests a taxi.)* *(The angry rather guests order a taxi ***after the party*** *.)*

Note: The original materials are in Dutch. The English translations are given in the parentheses below the original Dutch materials. Note that both literal and correct English translations are given for the answer sentences due to word order differences between Dutch and English. The syntactically incorrect sentences are marked by *. The critical words are underlined, and the linguistic focus is in boldface in the answers.

In addition to the factor Grammaticality, the factor Context (focus, non-focus) was manipulated by means of different wh-questions in the question-answer pairs. The wh-questions (who, what and when/where) inquired about specific information concerning different components of the answer sentences (subject NP, object NP and preposition respectively; see the questions in Table 1). Consequently, the syntactic violation occurring on the “subject nouns” was in focus position (in the who- question contexts in conditions 1, 2 and 3) or in non-focus position (in the when−/where- question contexts in the questions in conditions 4, 5 and 6). The violation occurred after the focus (e.g., “*Na afloop van het feest (after the party)*”) in the answer sentences in the when−/where- question contexts, while in the what- question contexts (e.g. *What does one order after the party?*, not presented in Table 1), the syntactic violation occurred in front of the focus (e.g., “*een taxi (a taxi)*”). Note that in the non-focus conditions, the questions contained pronouns such as *men (one)* rather than full nouns such as *gasten (guests)* to ensure that the critical words (e.g. *gasten*) in the answers were new information in both the non-focus and focus conditions. This was done since it has been shown that the information status (new vs. given) influences ERP responses to those words [19]. In addition, to make sure that the number agreement violation happened on the CWs (subject nouns) in the answer sentences, the verbs in the questions were used in singular forms, as generally the singular verbs in the questions can be followed by either plural or singular verbs in the answers.

In this way, a full factorial design was created with a combination of two variables: Grammaticality (Correct, Number agreement violation, Phrase structure violation) and Context (Focus, Non-focus), which created a total of six conditions: Focus/Correct, Focus/Number agreement violation, Focus/Phrase structure violation, Non-focus/Correct, Non-focus/Number agreement violation and Non-focus/Phrase structure violation. See Table 1 for examples of the materials.

We constructed 240 experimental items, each item participating in six conditions. The six conditions were distributed across six experimental lists through a Latin square procedure, with each list containing equal numbers of items per condition (40 items). In this way we made sure that all the items were presented in each list with no repetition of items. As a result, in each list, 120 items had CWs in focus position by using who- question contexts. Among the other 120 items, 60 items had CWs placed before the focus constituent by using what- question contexts, and the other 60 items had CWs located after the focus constituent by using where- question contexts (30 items) or when- question contexts (30 items).

In addition to the syntactic manipulation, we also included a semantic manipulation. However, since the focus of this paper is on the syntactic processing modulated by IS, we do not elaborate the semantic manipulations here. Four lists were built by assigning 40 items of each condition in one list (there were 160 items, four conditions per item), with no repetition of items within one list. Consequently, in each list, there were 40 items with a which-question, 40 items with a what-kind-of-question, 40 items with a where-question, and 40 items with a when-question. For each type of question, half of the answers contained a word that was semantically incongruent in relation to the question context, but never within the sentence itself.

Finally, 130 filler question-answer pairs were constructed to balance the correctness of the answers (fully congruent, syntactically incorrect, semantically incorrect) and the question types (who-, what-, when-, where-, which-, what-kind-of-), as well as to cover up the obvious difference between the syntactic and semantic manipulations. Among the fillers, 80 items were fully congruent, 40 of which served as fillers for syntactic materials. The answers contained a structure of “adjective + adverb + adjective + noun” to induce the subjects to take the noun as the violation point when they come across the structure of “adjective + adverb + noun” in the experimental materials. Another 40 fully congruent items served as fillers for semantic materials. There were also 50 filler items containing either syntactic violations (20 items) or semantic violations (30 items). The items with syntactic violation had similar question contexts as the semantic materials, while the items with semantic anomalies had similar question contexts as the syntactic materials, so that the subjects could not predict any syntactic or semantic violations by only reading the question contexts.

Twelve lists were built, in which the six lists of syntactic items were repeated twice, the four lists of semantic items were repeated three times, and the fillers were repeated twelve times. Each list comprised 240 experimental items, 160 additional items with a semantic manipulation, and 130 fillers. Each list was presented to two participants (one male and one female).

### Procedure

Subjects were seated in front of a computer monitor at approximately 80 cm distance. All the materials were presented in white fonts on a black background, with the font size of 27 for the whole questions and of 30 for the words of answers. A trial started with a fixation cross (duration 3000 ms) in the center of the screen, followed by a question that was presented as a whole sentence for 2500 ms. After a 100 ms black screen, the answer was presented word by word, with each word appearing for 300 ms, and an inter-stimulus interval (ISI) of 300 ms. The last word ended with a period. Three hundred milliseconds after the presentation of the last word, the next trial began. Participants were told not to move or blink when individual words appeared, but they were encouraged to blink during the presentation of the cross. There was no additional task other than to read for comprehension.

The materials in each list were arranged in a pseudorandom order, such that no more than three items of the same condition were presented in succession. The 530 items in one list were divided into 26 blocks (20 or 21 trials per block), with each block lasting about four minutes. In between each block there was a small break, after which subjects could start the beginning of the next block by pressing a button. The whole experiment was separated into two sessions (13 blocks per session). Each session took about two hours, including subject preparation, instructions and a short practice run consisting of 15 items. The subjects finished the two sessions on two different days, with a minimum of one day and a maximum of fourteen days in between both sessions. There is no influence of the delay on any one of the experimental conditions.

### EEG Recordings and Analysis

The EEG was recorded in an electromagnetically shielded cabin, with 60 surface active electrodes (Acticap, Brain Products, Herrsching, Germany) placed in an equidistant montage. The left mastoid electrode served as the reference, and a forehead electrode served as the ground. The vertical and horizontal eye movements were monitored by electrodes placed in the cap. All electrode impedances were kept below 20 KΩ during the experiment, which is well below what is recommended for active electrodes. EEG data were digitized at a rate of 500 Hz with a 100 Hz high cut-off filter and a 10 seconds time constant.

The EEG data were analyzed by the Brain Vision Analyzer software 1.05 (Brain Products). First, the data were re-referenced off-line to the average of both mastoids, then a band-pass filter of 0.5–30 Hz (48 dB/oct slope) was applied to the data. After that, the data of the two types of violations were separately segmented. For the number agreement violation condition, the critical epoch was defined from 150 ms before to 1200 ms after the onset of the CW, with baseline correction from 150 to 0 ms preceding word onset. For the phrase structure violation, the critical epoch started from 150 ms before the word preceding the critical word (CW-1) and lasted till 1200 ms after the onset of the CW (−150 to 1800 ms relative to the onset of CW-1), with the time window of 150 ms to 0 ms before the onset of CW-1 serving as the baseline. The epochs of the correct condition were defined in analogous ways as the two violation conditions: −150 to 1200 ms relative to the onset of CW as in the agreement violation condition, and −150 to 1800 ms relative to the onset of CW-1 as in the phrase structure violation condition. This was done for the following reasons. First, the phrase structure violations would elicit ERP effects for both the CW and for the CW-1 [10]. As a result, strong condition differences would already be present during the baseline interval of the analysis of the CW's for the phrase structure violation, which could be caused by an overlap with the P600 on the CW-1. This leads us to analyze both the CW and the CW-1 with a baseline correction from −150 to 0 ms relative to the onset of the CW-1. On the contrary, the number agreement violations would yield ERP effects only for the CW [10], which allows us to take the time window of −150 and 0 ms directly preceding CW as a baseline for this comparison. The necessity to select different baselines makes it difficult to directly compare the ERP amplitudes across the two types of violations. Second, the effects at CW-1 for the phrase structure violation were at a position where there was no syntactic violation in the grammatical sense, but only a violation of a syntactic preference. The effects are different from the ERP effects caused by a violation of syntactic constrains for the number agreement violation. In this sense, the observed ERP effects for the two types of violations were not directly comparable.

Then a semi-automatic artifact rejection procedure was applied. On average, 97% and 96% of all trials were kept, respectively, for the conditions with number agreement manipulation (including the number agreement violation condition and the correct condition) and the conditions with phrase structure manipulation (including the phrase structure violation condition and the correct condition). For statistical testing, trials were averaged in each condition for each electrode and each subject. In the end, two full factorial designs were tested, with each containing the two factors: Grammaticality (Syntactically correct: S+, Syntactically incorrect: S−) and Context (Focus: F+, Non-focus: F−).

### Statistical Analysis

Both on the basis of earlier studies [10], [11], [14] and visual inspection of the waveforms, we tested the statistical differences among conditions for two components: the standard N400 component in the latency window of 300–500 ms, and the later P600 component in the time window of 500–1200 ms. In addition, for the P600 elicited by the CW-1 in the phrase structure violation condition, a time window of 500–900 ms was selected.

The statistical significance of the difference between two conditions was evaluated by a cluster-based random permutation approach (see [20] for details on the method), which was implemented in the Matlab toolbox Fieldtrip [21]. This approach controls the Type-1 error rate in a situation involving multiple comparisons (one comparison for each of the 59 electrodes). Here is a brief description of the procedure. First, for every electrode a simple dependent-samples *t* test is performed. All adjacent electrodes exceeding a preset significance level (5% here) are grouped into clusters. For each cluster the sum of the *t* statistics is used in the cluster-level test statistic. Next, a null distribution which assumes no difference between conditions is created. This distribution is obtained by 1000 times randomly assigning the conditions in subjects and calculating the largest cluster-level statistic for each randomization. Finally, the actually observed cluster-level test statistics are compared against the null distribution, and clusters falling in the highest or lowest 2.5^th^ percentile are considered significant. We only reported the significant clusters in the results.

We examined the main effect of Grammaticality and Context, as well as their interaction, separately for the two types of violations. This procedure only allows for pair-wise comparisons. Therefore, the main effect of Grammaticality was acquired by comparing the amplitudes of S− conditions (the averaged amplitudes of F+S− and F−S− conditions) with that of S+ conditions (the averaged amplitudes of F+S+ and F−S+ conditions); similarly, the main effect of Context was obtained by comparing the amplitudes of F+ conditions (the averaged amplitudes of F+S+ and F+S− conditions) with that of F− conditions (the averaged amplitudes of F−S+ and F−S− conditions). Then the interaction between Grammaticality and Context was tested by comparing two subtractions: (F+S−)−(F+S+) versus (F−S−)−(F−S+). Since we have a strong a-priori hypothesis that IS has a modulation effect in language processing, we also performed planned comparisons on the syntactic violation effect for the focus and non-focus conditions separately. For each comparison, the averaged N400 or P600 amplitudes of all 59 electrodes were entered into the analysis.

## Results

We present the ERP results of the number agreement violation and the phrase structure violation separately in the following sections.

### Number Agreement Violation

**Figure 1:**
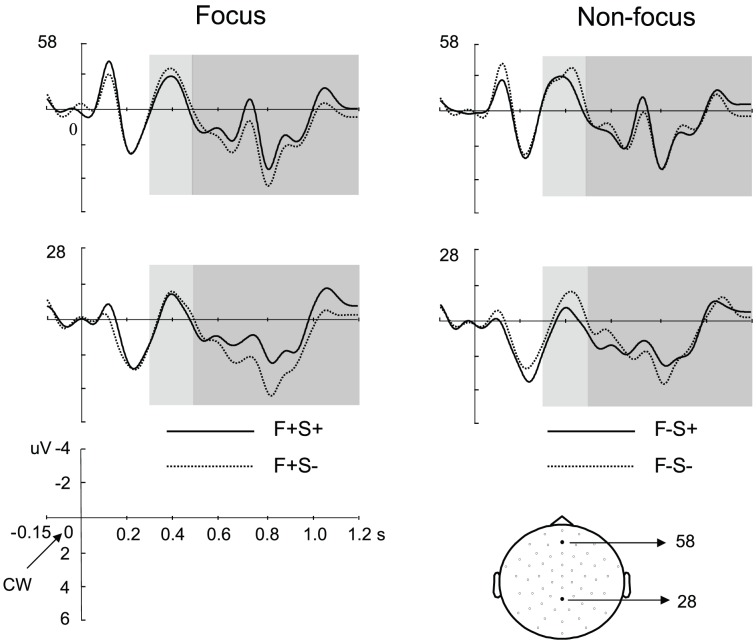
Grand averaoge waveforms for the number agreement violation and the correction condition. The waveforms are shown for the focus condition (left panel) and the non-focus condition (right panel), at two scalp sites (28, 58) which are indicated in the head model. The onset of the critical word (CW) is at zero. Negativity is plotted up. The N400 time windows (0.3–0.5 s) are marked by light gray boxes, while the P600 time windows (0.5–1.2 s) are marked by dark gray boxes. The selected posterior electrodes for statistical tests are painted in light gray on the head model. F+S+: Focus/Syntactically correct; F+S−: Focus/Number agreement violation; F−S+: Non-focus/Syntactically correct; F−S−: Non-focus/Number agreement violation.

Figure 1 shows the grand average waveforms evoked by the CW of the agreement violation and the correct condition.

In the N400 time window, the agreement violation elicited a larger N400 amplitude than the correct condition over the central region (main effect for Grammaticality: p = .01; mean amplitudes of S− vs. S+ over the electrodes showing the significant effect: −2.42 µV vs. −1.21 µV). The N400 effect was not modulated by Context, as indicated by the absence of a significant interaction between Grammaticality and Context (no significant cluster). Also the main effect of Context failed to reach significance (no significant cluster).

For the P600 component, the agreement violation evoked larger P600 amplitude than the correct condition over the right posterior region (main effect for Grammaticality, p = .043; mean amplitudes of S− vs. S+: 2.95 µV vs. 1.91 µV). No effect of Context was found (main effect for Context: no significant cluster). Importantly, however, the P600 effect was different between the focus and non-focus conditions, as revealed by a marginally significant interaction between Grammaticality and Context (p = .067). Although the interaction effect is only marginally significant, given our strong a-priori hypothesis that IS modulates language processing, we tested the syntactic violation effect for the focus and non-focus conditions separately. The planned comparisons revealed that a significant P600 effect was only elicited in the focus condition over the central-posterior region (p<.001; mean amplitudes of F+S− vs. F+S+: 1.40 µV vs. 0.67 µV), but not in the non-focus condition (no significant cluster).

### Phrase Structure Violation

**Figure 2:**
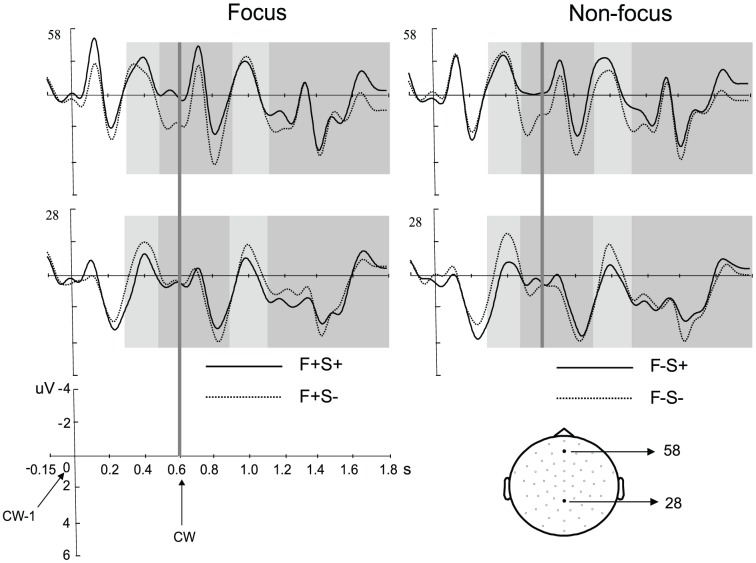
Grand average waveforms for the phrase structure violation condition and the correct condition. The waveforms are shown for the focus condition (left panel) and the non-focus condition (right panel), at two scalp sites (28, 58) which are indicated in the head model. The onset of the word preceding the CW (that is, CW-1) is at zero, and the onset of the CW is at 0.6 s, marked by the light gray line. Negativity is plotted up. The N400 time windows (0.3–0.5 s, 0.9–1.1 s) are marked by light gray boxes, while the P600 time windows (0.5–0.9 s, 1.1–1.8 s) are marked by dark gray boxes. The selected posterior and anterior electrodes for statistical tests are respectively painted in light gray and dark gray on the head model. F+S+: Focus/Syntactically correct, F+S−: Focus/Phrase structure violation, F−S+: Non-focus/Syntactically correct, and F−S−: Non-focus/Phrase structure violation. CW: critical word.

Figure 2 displays the grand average waveforms evoked by the CW as well as the word preceding the CW (CW-1) for the phrase structure violation and the correct condition.

We observed a significantly larger N400 for the violation condition than for the correct condition over the central-posterior region for both the CW (p = .005; S− vs. S+: −1.32 µV vs. 0.25 µV) and the CW-1 (p = .007; S− vs. S+: −1.86 µV vs. 0.07 µV), whereas the main effect of Context as well as the interaction were not significant (all p values >.10).

For the statistical analysis of the P600, the phrase structure violation condition elicited a larger positivity than the correct condition only for the CW-1 over the anterior region (p = .01; S− vs. S+: 2.20 µV vs. 0.47 µV), but not for the CW (p = .18). No interaction effect was found for either the CW or the CW-1 (no significant cluster). Planned comparisons on the syntactic violation effect revealed significant P600 effects for the CW-1 in both the focus (p = .017; F+S− vs. F+S+: 1.18 µV vs. 0.22 µV) and non-focus (p = .007; F−S− vs. F−S+: 1.16 µV vs. 0.23 µV) conditions, but not for the CW in either the focus (no significant cluster) or the non-focus (p = .09) condition. The failure to find a significant positive effect for the CW might be explained by the conservativeness of the statistical analysis in detecting relatively less robust effects. Based on visual inspection, we performed a more sensitive statistical test. The amplitude values in the P600 latency interval (500–1200 ms) in the four frontal electrodes that show the largest positive effects were averaged per condition for each subject. Then an ANOVA was performed on the mean values, with the factors Grammaticality (Correct, Phrase structure violation) and Context (Focus, Non-focus). The results did reveal that the phrase structure violation condition evoked a larger positivity than the correct condition (F_(1,23)_ = 4.39, p = .047), while no main effect of Context, nor any interaction with Context was found (all Fs_(1,23)_<1).

**Figure 3:**
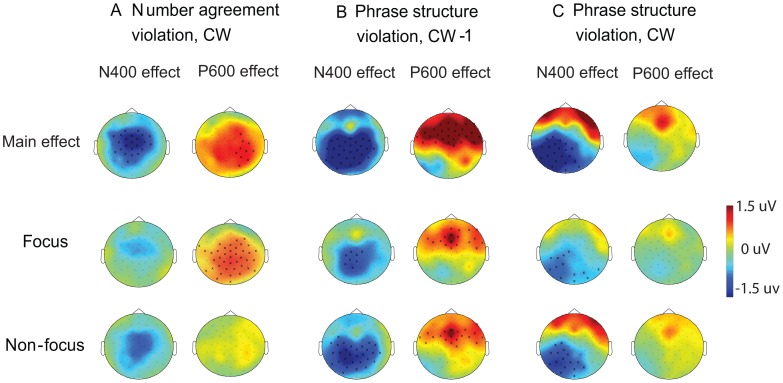
Topographies of the ERP effects for the number agreement violations and the phrase structure violations. They were computed from values resulting from the subtractions of: A. ERPs for the correct condition from that for the number agreement violation condition in the N400 and P600 time windows evoked by the CW; B. ERPs for the correct condition from that for the phrase structure violation condition in the N400 and P600 time windows evoked by the CW-1; C. ERPs for the correct condition from that for the phrase structure violation condition in the N400 and P600 time windows elicited by the CW. CW: critical word. CW-1: the word preceding the critical word. The three rows show the averaged as well as the separate effects of Grammaticality for the focus and non-focus conditions. The electrodes that showed significant effects were marked by “x”.

Figure 3 displays the scalp distribution of all the ERP effects. To clearly illustrate the violation effects as well as the IS modulations on the effects, we present both the main effects of the Grammaticality, as well as the violation effects separately for the focus and non-focus conditions. The number and locations of the electrodes in each significant cluster can also be seen in Figure 3, where the electrodes that show significant effects were marked by “x”. Note that although there is a significant main effect of the N400 component for the number agreement violation, no significant N400 effect was revealed when it was tested separately for the focus and non-focus conditions. This might be due to the limited statistical power in detecting the subtle N400 effects when a small number of trials were averaged.

### Post-hoc Cloze Probability Test

We observed N400 effects for both types of syntactic violations. The N400 effect elicited by a phrase structure violation has been reported in other studies [9], [10], [11]. It has been related to the semantic consequences of the severe syntactic violation. However, the subtle number agreement violation also evoked an N400 effect although it seems not to exert much influence on semantic unification. We speculated that the verbs with plural (*order*) and singular (*orders*) forms in the correct and violation conditions respectively, might generate different semantic constraints with regard to the upcoming subject NP, which results in different semantic predictions. For instance, as in the example of our experimental item, the semantic association between *order* and *guests* is stronger than that of *orders* and *guest*, so the verb with a plural form (*order*) is more likely to be followed by the lemma *guest* comparing to the verb with a singular form (*orders*). Such potential differences in semantic probability can be verified by a cloze probability test, in which subjects completed the sentences with the CWs omitted. Note that we took the lemma instead of the exact words the subjects chose into calculation, so that the cloze probability reflects the predictability of the subject NP at the lemma level. Therefore, we measured the cloze probability of the lemma regardless of the number features in the four conditions (Focus/Correct, Focus/Number agreement violation, Non-focus/Correct, Non-focus/Number agreement violation) in another 40 subjects who did not participate in the EEG experiment. An ANOVA analysis (2 Grammaticality × 2 Context) performed on the cloze probability ratings revealed lower cloze probability for the number agreement violation condition (the percentage of Mean ± SD is 9.26±5.69) than for the correct condition (the percentage of Mean ± SD is 11.02±3.47), which was confirmed by the main effect of Grammaticality (F_(1,39)_ = 4.17, p = .048). Besides, the cloze probability test showed no main effect of Context (F_(1,39)_ = .26, p = .61), nor was the interaction between Context and Grammaticality (F_(1,39)_ = 3.05, p = .09) significant.

## Discussion

We examined how Information Structure (IS) modulates the level of syntactic analysis during online language processing. ERP responses to number agreement violations and to phrase structure violations were recorded in focus and non-focus conditions. For the (relatively subtle) number agreement violation, we found a P600 effect in the focus condition but not in the non-focus condition. For the (more salient) phrase structure violation, comparable P600 effects (although frontally distributed) were found between the focus and non-focus conditions. In addition to the P600 effect, both types of syntactic violations elicited N400 effects. We discuss the results in more detail below.

### IS and P600 Effects in Response to Syntactic Violations

The number agreement violation produced different ERP responses for the focus and non-focus conditions. A P600 effect was evoked when the violation occurred in focus position, while no significant P600 effect was found when it was in non-focus position. A P600 effect in response to number agreement violations has been reported in many other studies [10], [11], [22], [23]. It has been interpreted as reflecting the difficulty of syntactic unification in the presence of inconsistent number features on subject and verb forms, or alternatively, as reflecting the detecting of the error [24]. Crucially, the lack of a P600 effect in the non-focus condition is in line with our hypothesis that comprehenders engage in shallow processing for non-focused linguistic input, which supports our notion that IS modulates the depth of processing not only at the level of semantics (as shown in previous studies), but also at the level of syntactic analysis.

The results confirm the claim that focused information receives more attentional resources and is more deeply processed [4], [5], [7]. In our previous ERP studies [12], [13], we found that in response to semantic violations, a larger N400 effect was evoked when the eliciting word was in focus compared to in non-focus position. The current results are consistent with these studies by showing that a number agreement violation elicited a larger P600 effect for focused information than for non-focused information. In all three studies, the question context generated a prediction as to where the new information would appear in the answer sentence. Therefore, people might have allocated more attentional resources to this focus position. Hence, the focused information was processed thoroughly. Fewer resources might have been allocated to the non-focused information, resulting in less detailed processing, both at the semantic and at the syntactic level.

Nevertheless, a very salient syntactic violation, such as a phrase structure violation, is not sensitive to the modulatory influence of IS: in both the focus and non-focus conditions, we observed similar P600 effects in response to the phrase structure violation compared with the correct condition for both the critical word and the word preceding the critical word. This suggests that the influence of IS is overridden when a syntactic preference is very salient. In the present study, although IS directed more attentional resources towards the focused information than towards the non-focused information, the prominent phrase structure violation in non-focus position might have captured attention immediately, eliciting a P600 effect similar to that in the focus condition.

The P600 effect elicited by the word preceding the critical word confirm previous findings that comprehenders quickly assign a preferred structure based on the frequency of alternative syntactic constructions, or on the basis of some computational economy principle [10], [11]. Note that here, the P600 effects were frontally distributed, which is often associated with the processing costs involved in overriding the preferred or most activated syntactic structure [10], [25], [26]. The anterior positivity has also been found to be triggered by expectancy violations even without any syntactic incongruence [27], [28], giving rise to the speculation that the anterior positivity is a processing consequence of unexpected input in general. In this sense, one might argue that the absence of IS influence on the anterior P600 effects might simply reflect the lack of IS modulation on the predictability of the CWs rather than on syntactic processing. However, the current study specifically manipulated the syntactic structure of the input, and it is well established that syntactic violations can drive P600 effects, sometimes also with an anterior distribution [10], [25], [26]. Although it is difficult to disentangle the two accounts of the anterior P600 effect, the experimental manipulation strongly suggests that syntactic processing is not modulated by IS when a violation is very salient.

Overall, the divergent results between the number agreement violation and the phrase structure violation suggest that the role of IS in modulating the depth of processing during language comprehension depends on the salience of the information. In general, IS is sufficiently powerful to play a role by modulating attentional resources in a top-down manner, with shallow processing occurring to non-focused information. But if information is very salient (e.g. because it violates a strong syntactic prediction), it can override the top-down control of IS, giving rise to the same extent of processing for focused and non-focused information.

### A “Good-enough” Approach during Language Processing

The absence of a P600 effect in response to the number agreement violation in the non-focus condition is compatible with a “good-enough” account of language comprehension. This position claims that people sometimes engage in shallow processing and achieve incomplete representations (for reviews see [29], [30], [31], [32], [33]). Several behavioral studies have provided evidences for a “good-enough” syntactic processing account. For example, Ferreira [34] asked subjects to name the agent of the action for passive sentences such as *The dog was bitten by the man.* She found that a large proportion of subjects wrongly took *the dog* as the agent, which suggested that parsing was guided by heuristically assigning the first NP as the agent of the action. The selection of the heuristic might be due both to the fact that the first noun in a sentence is usually the agent of the action, and to the world knowledge that it is normally a dog who bites a man rather than the opposite. Two other studies [35], [36] examined the effect of a missing verb phrase in sentences with double centre-embedded structures such as *The Mexican meal/that the gastronomic critic/that the journal hired/tasted in the new restaurant* ***/*** *had a strange smell.* They found that subjects rated the sentence where the second verb phrase was omitted (*tasted in the new restaurant*) as easier to understand than the grammatical sentence. Nevertheless, these studies employed sentence with complex syntactic structure, which makes it unclear whether the results are caused by a good-enough strategy, or whether they are due to poor comprehension. In addition, they all used off-line tasks, which provide a relatively indirect index of the underlying cognitive processes. By using ERPs, our findings further strengthen the results of these behavioral studies [34], [35], [36]. Therefore, the available data support a “good-enough” processing strategy during language comprehension at the level of both semantics and syntax [29], [30], [31], [32], [33].

### N400 Effects Evoked by Syntactic Violations

In addition to P600 effects, we also observed N400 effects in response to syntactic violations. For the phrase structure violation, the severe syntactic violation has immediate consequences for the semantic unification of the words into a coherent message-level representation [9], [10], [11]. This semantic unification difficulty elicits an N400 effect [8].

However, the subtle number agreement violation also evoked an N400 effect. The cloze probability test showed that the critical words in the number agreement violation condition had a lower cloze probability than in the correct condition. It has been shown that the difference in cloze probability affects the size of N400 amplitudes (for a review, see [8]). Therefore, the N400 effect elicited by the number agreement violations can be attributed to the fact that the agreement violations altered the predictability of the critical words, e.g. the verbs with plural and singular forms in the correct and violation conditions respectively created different semantic predictions with regard to the upcoming subject nouns. Moreover, the N400 effects did not differ between the focus and non-focus conditions, which indicates that IS did not have an effect on the predictability of the CW.

The presence the N400 effects further supports our claim that IS plays a role in modulating syntactic processing: one could argue that the lack of a P600 effect in the non-focus condition might simply reflect the fact that the number agreement violation has not been detected (it being out of focus) due to the orthographic similarity between the correct and violation conditions. However, the presence of an N400 effect in the non-focus condition demonstrates that the subjects were able to extract at least the semantic consequences of number marking and tried to integrate this information into the context.

It appears that the comparable N400 effects between the focus and non-focus conditions are in contrast to the two previous studies where the N400 effect elicited by semantic violations was larger in the focus than in the non-focus condition [12], [13]. The lack of IS modulation on the N400 effects in the current study appears to be contradictory to the role of IS in modulating language processing. However, the syntactic manipulation in the current study is quite different from previous semantic manipulations. First, the phrase structure violation is so salient that IS markings do not override the violation effect. Therefore, we did not observe a modulation of the P600 and N400 effects. For the agreement violation, the unexpected N400 effect was found to be caused by a subtle difference in the cloze probability (around 2%). This N400 effect is smaller than usually observed for a semantic anomaly. Hence, the effect might have been too subtle to result in an IS modulation. Furthermore, an additional possibility is a temporal overlap between N400 and P600 effects. Due to the reversed polarity between the P600 and N400 components, the strong P600 effect elicited in the focus condition might have masked the presence of an N400 modulation by IS.

However, these explanations are somewhat speculative, and require further testing in future studies.

### Conclusions

By examining ERP responses to number agreement violations and phrase structure violations in both the focus and non-focus conditions, we have provided evidence for the influence of IS on the depth of syntactic processing. We found that number agreement violations elicited a P600 effect for the focus condition but not for the non-focus condition, while the phrase structure violation elicited P600 effects for both the focus and non-focus conditions. These results indicate that IS modulates not only the depth of semantic analysis (the Moses illusion, see [37]), but importantly also the depth of syntactic processing. Chomsky has argued that syntax is the core of the human language faculty, playing a crucial role in arriving at a semantic interpretation [38]. Here we have provided evidence that semantic interpretations might result in the absence of fully processing all the syntactic features in the input. Therefore, we label the effect the Chomsky illusion. This is presumably due to the possibility that in a “good-enough” framework, focused information recruits more attentional resources than non-focused information. The influence of IS is however overridden by very salient syntactic information, indicating that IS plays a subtle role in modulating resource allocation during language comprehension.
